# Tac1 deficiency reduces the severity of enteric bacterial infection

**DOI:** 10.1371/journal.ppat.1014587

**Published:** 2026-09-18

**Authors:** Elliot Lloyd, Michael Cremin, Emmy Tay, Kristina Sanchez, Jungjae Park, YuanYuan Lei, Melanie G. Gareau, Colin Reardon

**Affiliations:** Department of Anatomy, Physiology & Cell Biology. School of Veterinary Medicine, University of California Davis, Davis, California. United States of America; National Institute of Pharmaceutical Education and Research Hyderabad, INDIA

## Abstract

**Background:**

Infection with enteric bacterial pathogens continues to cause significant morbidity and mortality throughout the world. Effective control of these infections requires coordinated immune responses that are shaped in part by bidirectional communication between the nervous and immune systems. Sensory neurons are now well appreciated to detect inflammatory or microbial stimuli in numerous organ systems, including the gastrointestinal (GI) tract, and for eliciting the recruitment or activation of specific immune cells. Central to the host protective effect of these neuroimmune circuits is the localized release of neurotransmitters that activate substance P (SP) receptor signaling. However, the precise contribution of sensory neuropeptides encoded by the *Tac1* gene, such as SP, in the host response to enteric infection remains incompletely understood.

**Methods:**

The role of the Tac1-encoded sensory neurotransmitters in the host response to *Citrobacter rodentium* was assessed using *Tac1*^*-/-*^ and wildtype (WT) mice. Infection and inflammation were determined by bacterial enumeration, histopathology, qPCR, and flow cytometry.

**Results:**

*Tac1*^-/-^ mice had significantly reduced pathogen shedding and colonic bacterial burden, accompanied by decreased expression of inflammatory cytokines and chemokines compared to WT controls. In accordance with reduced chemokine production, we observed reduced colonic recruitment of neutrophils, monocytes, and IFNγ- and IL-17A-producing T-cells in *Tac1*^*-/-*^ compared with WT mice. This reduced immune response was associated with markedly reduced colonic histopathology.

**Conclusions:**

Sensory neuropeptides encoded by *Tac1* regulate key aspects of the immune response to enteric bacterial infection and may serve as unique targets in the treatment of enteric disease.

## Author summary

Intestinal infections remain a major cause of illness and death worldwide. Nerves that detect tissue injury, microbes, or microbial products can shape the immune response by releasing chemical messengers. The *Tac1* gene encodes several of these messengers, including substance P, but their role during intestinal bacterial infection remains unclear. To address this, we infected mice lacking the *Tac1* gene with the mouse-adapted bacterial pathogen *Citrobacter rodentium*. Mice deficient in *Tac1* had fewer bacteria than wild-type mice and exhibited reduced inflammation. This included reduced recruitment of neutrophils, monocytes, and T cells to the colon. Colonic tissue damage was also reduced. Our findings show that Tac1-derived neuropeptides do not simply enhance antibacterial immunity but can amplify inflammation without improving pathogen control. These results highlight that many signals contribute to the host immune response and that selective targeting of this pathway could provide a new approach to limiting disease during infection.

## Introduction

The ability to mount immune responses to pathogens while limiting the potential for aberrant or excessive inflammation that can harm the host is critical to protection. In the gastrointestinal (GI) tract, the mucosal immune system distinguishes between noxious stimuli, pathogens, and dietary and commensal microbiota-derived substances. The mouse-adapted pathogen, *Citrobacter rodentium* has greatly aided our understanding of host defense in the gut*,* revealing unique roles of innate lymphoid cells (ILC), tissue-resident macrophages, the recruitment of neutrophils, monocytes, and eventually T-cells after oral infection [1–5]. These immune mechanisms operate in a coordinated manner throughout infection, culminating in pathogen eradication through B-cell-produced immunoglobulins. Although cytokines and chemokines produced by immune and stromal cells have been well established in this coordinated defense, signals exchanged between these cells and the nervous system can also alter the trajectory of an immune response, and thus the course of disease [6,7].

The GI tract is replete with innervation, including the enteric nervous system (ENS) that is intrinsic to the intestine, and extrinsic innervation, which originates in the dorsal root ganglia and sympathetic chain ganglia [8]. While the ENS controls many physiological functions of the GI tract, extrinsic innervation can also influence these processes [9]. Detection of noxious stimuli such as excessive heat, inflammatory products, or bacterial components can be achieved by specialized sensory neurons expressing the polymodal nociceptor Transient Receptor Potential Vanilloid 1 (TRPV1) [10,11]. Activation of TRPV1, and consequently these neurons, can cause the localized release of peptide neurotransmitters such as substance P (SP) [12–14]. This neuropeptide is encoded by the tachykinin precursor gene *Tac1*, which gives rise to multiple tachykinin neuropeptides, including neurokinin A. During enteric bacterial infection, we and others have demonstrated that TRPV1+ neurons coordinate the host immune response [15,16]. We have also shown that SP receptor signaling in distinct immune and stromal populations fine-tunes the host response, modulating T-cell recruitment and IFNγ production [17]. These findings are consistent with the defined roles of this neurotransmitter during neurogenic inflammation, where SP increases blood vessel dilation and permeability and aids the recruitment of immune cells by increasing endothelial cell expression of adhesion molecules [18–20]. It is also now appreciated that SP can exert biological effects, such as mast cell degranulation, through activation of Mas-related G protein receptors [21]. As a result, the biological effect of SP in the GI tract is of considerably greater complexity than previously appreciated. This complexity extends to the source of SP in the intestine, with expression in extrinsic sensory neurons, putative interneurons, motor neurons of the ENS [22,23], and in enteroendocrine cells [24–26]. The unique positioning of these cells in the GI tract, coupled with the potential for SP-mediated activation of responding cells via non-canonical receptor types, suggests diverse roles for SP in regulating host responses.

Based on these observations, we hypothesized that loss of Tac1 gene products, such as substance P, would alter the response to enteric bacterial infection with *C. rodentium*. Consistent with a role for Tac1 during infection, *Tac1*^*-/-*^ mice exhibited significantly reduced fecal bacterial shedding, colonic bacterial burden, and infection-induced colonic histopathology compared with wildtype (WT) mice. Although SP is a known secretagogue in the colon, Ussing chamber analysis did not identify any defect in baseline and induced ion secretion, conductance, and permeability in *Tac1*^*-/-*^ mice. In assessing innate and adaptive immune responses during infection, significant reductions in Tumor Necrosis Factor alpha (TNFα) and select chemokines that recruit neutrophils and T cells were observed in *Tac1*^*-/-*^ mice compared with WT. Significantly reduced Interferon-gamma (IFNγ), and expression of IFNγ-dependent genes such as inducible nitric oxide synthase (*Nos2*), were also found in infected *Tac1*^*-/-*^ compared to WT mice. In addition to the reduction in colonic T-cells, intracellular cytokine staining of lamina propria T-cells revealed significantly reduced IFNγ and IL-17A-producing CD4 + T-cells in infected *Tac1*^*-/-*^ compared to WT mice. With the reduction in specific T-cell responses*, in vitro* Th1 differentiation was performed, revealing no intrinsic defects in generating these responses. Together, these data demonstrate that *Tac1*-derived neuropeptides coordinate specific aspects of the host response to the enteric bacterial pathogen *C. rodentium*.

## Methods

### Ethics statement

The University of California, Davis Institutional Animal Care and Use Committee (IACUC) reviewed and approved all procedures involving vertebrate animals (protocol number 24946).

### Animals

C57BL/6J and *Tac1 − / −* mice were obtained from Jackson Laboratories and bred at UC Davis. Male and female mice (7–12 weeks) were gavaged with LB or *C. rodentium* (DBS100, 10⁸ CFU). Samples were collected at 4, 10, 14, and 30 days post-infection (dpi). Distal colonic motility was assessed by fecal pellet output over 20 minutes. Bacterial burden was quantified from feces or distal colon by homogenization and plating on MacConkey agar. In select experiments, mice received vehicle or the MrgprA3 antagonist dictamnine (2.5 mg/kg, i.p., daily, Cayman Chemical, Ann Arbor, MI) starting at infection [27]. All animals had *ad libitum* access to food and water.

### Quantitative real-time PCR

Colon tissues were collected in TRIzol, homogenized, and RNA was extracted per manufacturer’s instructions (Invitrogen, Carlsbad, CA). cDNA synthesis was performed using iSCRIPT (Bio-Rad, Hercules, CA), followed by qPCR using indicated primers (Table 1) [28] on a QuantStudio6 platform.

**Table 1:** Primer sequences.

Target	Forward 5’-3’	Reverse 5’-3’
*Il-1β*	CTGTGACTCAT GGGATGATGATG	CGGAGCCTGTAGTGCAGTTG
*Il-6*	TAGTCCTTCCTACCCCAATTTCC	TTGGTCCTTAGCCACTCCTTC
*Il-17a*	TTTAACTCCCTTGGCGCAAAA	CTTTCCCTCCGCATTGACAC
*Il-22*	ATGAGTTTTTCCCTTATGGGGAC	CTGGAAGTTGGACACCTCAA
*Ifnγ*	GCCACGGCACAGTCATTGA	TGCTGATGGCCTGATTGTCTT
*Tnfa*	CCCTCACACTCAGATCATCTTCT	GCTACGACGTGGGCTACAG
*Nos2*	GTTCTCAGCCCAACAATACAAGA’	GTGGACGGGTCGATGTCAC
*Regiiiγ*	CCTCAGGACATCTTGTGTC	TCCACCTCTGTTGGGTTCA
*β-actin*	GGCTGTATTCCCCTCCATCG	CCAGTTGGTAACAATGCCATGT
*Cxcl1*	TCCAGAGCTTGAAGGTGTTGCC	AACCAAGGGAGCTTCAGGGTC
*Cxcl2*	CTCTCAAGGGCGGTCAAAAAGTT	TCAGACAGCGAGGCACATCAGGTA
*Cxcl3*	CATCCAGAGCTTGACGGTGA	ACACATCCAGACACCGTTGG
*Cxcl6*	TGGATCCAGAAGCTCCTGTGA	TGCATTCCGCTTAGCTTTCTTT
*Cxcl9*	GCAGTGTGGAGTTCGAGGAACCC	CCGAGTCCGGATCTAGGCAGGT
*Cxcl10*	CCAAGTGCTGCCGTCATTTTC	GGCTCGCAGGGATGATTTCAA
*CCL2*	TTAAAACCTGGATCGGAACCAA	GCATTAGCTTCAGATTTACGGGT
*Nox1*	CCTCCTGACTGTGCCAAAGG	ATTTGAACAACAGCACTCACCAA
*IL12p40*	TGGTTTGCCATCGTTTTGCTG	ACAGGTGAGGTTCACTGTTTCT
*Tac1*	ATTCCTTTGTTGGACTAATGGGC	AGCTCTTCTTTCGTAGTTCTGC

### *In vitro* T-cell differentiation and ELISA

CD4 ⁺ T cells were isolated from mesenteric lymph nodes by negative selection using an iMAG system (BD Biosciences, Franklin Lakes, NJ). Cells (25,000/well) were cultured on plate-bound anti-CD3 (Cat# 40–0032 Tonbo Biosciences, San Diego, CA) with or without anti-CD28 (Cat# 40–0281 Tonbo Biosciences) under Th1-polarizing conditions (anti–IL-4 Cat# 16–7041 Invitrogen, IL-2 Cat# 212–12 and IL-12 Cat# 210–12 Peprotech Cranbury, NJ). After 96 h, cells were restimulated with Cell Stimulation Cocktail (phorbol 12-myristate 13-acetate, eBioscience, San Diego, CA), and IFN𝛾 in supernatants was quantified by ELISA according to manufacturer’s instructions (Cat# 88-7314-22, Invitrogen).

Assay of total and *Citrobacter rodentium* reactive immunoglobulins was conducted by growing *Citrobacter rodentium* in Luria Broth, followed by centrifugation and resuspension in 1X PBS with protease inhibitor cocktail (Sigma-Aldrich, St. Louis, MO; 4693116001), and sonication on ice (6 × 30s). Lysates were centrifuged (15,000 × g, 15 min), and supernatants were coated onto high-binding 96-well plates (2 µg/mL, overnight, 4°C). Plates were blocked with 1% BSA (2 h, room temperature), then incubated with sera serially diluted twofold in 1% BSA, starting at 1:50 (IgG1, IgG2b, IgG2c, IgG3) or 1:5 (IgA, IgM) for 2 h at 37°C. HRP-conjugated anti-mouse secondary antibodies (1:400; Southern Biotech, Birmingham, AL; 5300–05) were applied for 2 h at 37°C, followed by TMB substrate and stop solution (2N H2SO4). Absorbance was read at 450 nm with background subtraction at 570 nm. Titers were expressed as OD450 versus the reciprocal of the log2 dilution. Group comparisons used two-way ANOVA with Geisser-Greenhouse correction and Sidak’s multiple comparisons test (GraphPad Prism 9.0), with significance set at p < 0.05.

### Lamina propria lymphocyte isolation

Colons were processed to remove epithelial cells, followed by enzymatic dissociation of the lamina propria using a commercial kit (Miltenyi Biotec, Gaithersburg, MD). Single-cell suspensions were filtered, washed, and used for downstream analyses.

### Flow cytometry

Cells were stained using standard flow cytometry protocols, including Fc blocking, viability staining, surface marker staining (Table 2), fixation, and permeabilization where appropriate. For intracellular cytokine detection, cells were stimulated with Cell Stimulation Cocktail (eBioscience) in the presence of GolgiPlug (1:500, Cat# 555029, BD Biosciences) prior to staining for IFN𝛾 (cat# 25-7311-82, Invitrogen) and IL-17A (cat# 561020, BD Biosciences). Data were acquired on an LSRII cytometer and analyzed using FlowJo.

**Table 2:** Flow cytometry and T-cell isolation antibodies.

Target	Clone	Fluorophore	Manufacture	Catalog Number
Ter119	Ter119	Biotin	Tonbo Biosciences	30-5921-U500
CD8a	53-6.7	Biotin	Tonbo Biosciences	30-0081-U500
Cd11b	M1/70	Biotin	Tonbo Biosciences	30-0112-U500
Cd11c	N418	Biotin	Tonbo Biosciences	30-0114-U500
B220	RA3-6B2	Biotin	Tonbo Biosciences	30-0452-U100
CD317	eBio927	Biotin	Invitrogen	13-3172-82
NK1.1	PK136	Biotin	Tonbo Biosciences	30-5941-U100
CD45	30-FF1	efluor450	eBioscience	48-0451-82
CD3	145-2C11	APC	Tonbo Biosciences	20-0031-U100
CD4	V4	AF700	Biolegend	100430
CD4	RM4–5	FITC	BD Pharmogen	553047
CD64	REA286	PE	Miltenyi Biotec	130-118-684
Ly6G	1A8	FITC	Tonbo Biosciences	35-1276-U100
Ly6C	HK1.4	PE-Cy7	Invitrogen	25-5932-82
gp38	8.1.1	AF488	Biolegend	127405
CD31	MEC13.3	APC/Cyanine7	Biolegend	102533
IL17a	XMG1.2	PE-Cy7	Invitrogen	25-7311-82
IFN γ	TC11-18H10.1	PE	Biolegend	506903

### Determining *Tac1* expression on sorted colonic cells

Lamina propria cells from LB-treated or *C. rodentium*–infected mice (10 dpi) were subjected to Fluorescence-Activated Cell Sorting (FACS) to isolate endothelial, T cell, and neutrophil populations. RNA was processed using a direct RT-qPCR kit (Takara), and Tac1 expression was quantified using PrimerBank primers. Intestinal epithelial cells were isolated by EDTA dissociation. RNA extracted from the Dorsal root ganglia was used as a positive control, and data were normalized to these samples.

### Ussing chambers analysis of intestinal physiology function

Studies were conducted as described previously. In brief, segments of distal ileum were collected and immediately placed in cold oxygenated Ringer’s buffer (pH 7.35 ± 0.02) containing (in mM): 115 NaCl, 25 NaHCO_3_, 1.2 MgCl_2_, 1.25 CaCl_2_, and 2.0 KH_2_PO_4_. Intestinal sections were cut open along the mesenteric border, gently rinsed, and mounted in Ussing chambers (Physiologic Instruments, San Diego, CA), exposing 0.1 cm^2^ of epithelial surface area to Ringer’s buffer at 37 °C with Glucose (10 mM) on the serosal side of the chamber for metabolic support, and an equimolar concentration of mannitol (10 mM) on the mucosal side. Agar-salt bridge electrodes connected to a voltage-clamp measured transepithelial short-circuit current (Isc [μA/cm^2^]), an indicator of active ion transport, and the potential difference after a 15-minute equilibration period, using Acquire & Analyze II software (Physiologic Instruments). Transepithelial conductance (G [mS/cm^2^]), was calculated as an indicator of tight junction ionic permeability. Macromolecular permeability was assessed by adding 400 μg/mL FITC–dextran (4 kDa, Sigma-Aldrich) to the mucosal chamber and serosal samples (200 μl) taken every 30 minutes for 2 hours, and FITC concentrations were quantified using a fluorescence plate reader (Synergy H1, BioTek Instruments, Inc).

### Statistics

Data were analyzed using Prism 10.0 with Student’s t test or one- or two-way ANOVA with Tukey’s post hoc testing. Data are presented as mean ± SEM.

## Results

### Reduced *C. rodentium* bacterial burden and histopathology in *Tac1*^*-/-*^ mice

**Fig 1:**
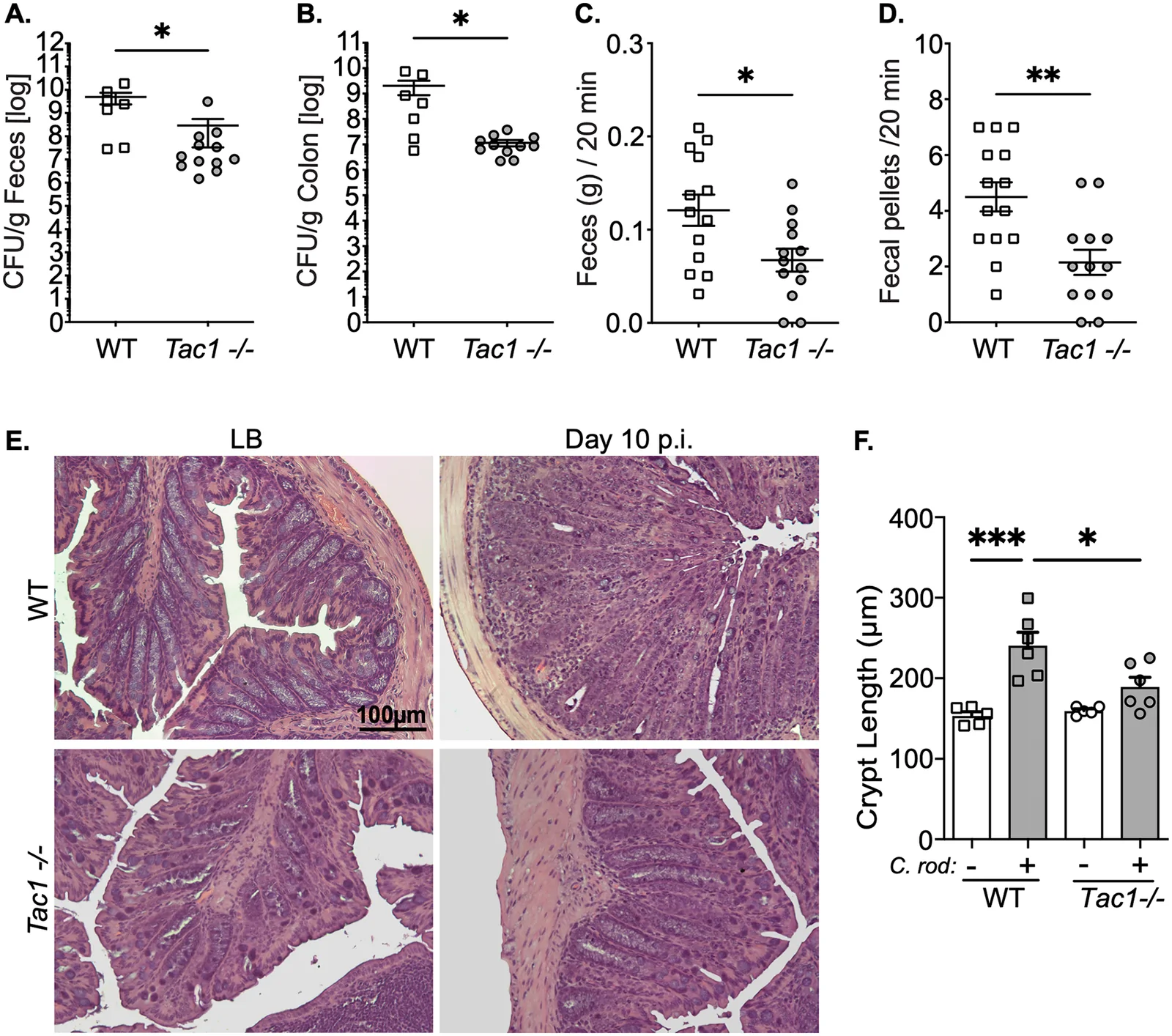
Reduced *C. rodentium* burden and histopathology in Tac1^-/-^ mice. Quantification of *C. rodentium* bacterial infection in the feces **(A)** and the colonic tissue **(B)** of wildtype (WT) and *Tac1*^*-/-*^ mice 10 days post-infection. Distal colonic motility assessed by the weight of fecal output **(C)** and the number of pellets **(D)**. Colonic histopathology was assessed in H&E sections **(E)**, with quantification of crypt length **(F)**. Data presented from individual mice with mean ± SEM, *P < 0.05, ** P < 0.005, ***P < 0.001. Student’s two-tailed t-test **(A-D)**, ANOVA followed by post-hoc analysis with Tukey’s multiple comparison test **(F)**. scale bar = 100 µm.

Infection with *C. rodentium* was significantly reduced, as indicated by fecal bacterial shedding (Fig 1A), and colonic adherent bacteria (Fig 1B) in *Tac1*^*-/-*^ mice compared to WT controls 10 days post-infection (dpi). Further assessment of the course of bacterial infection in subsequent cohorts of mice revealed no change in bacterial burden early in the infection (4 dpi), with significantly reduced fecal CFU at 14 dpi, and no effect on bacterial clearance in *Tac1*^*-/-*^ compared to WT mice (30 dpi, S1A Fig). Given the established role of SP in regulating colonic motility, we evaluated distal colonic motor function by measuring fecal output in uninfected mice. As expected, there was a significant reduction in the weight (Fig 1C) and number (Fig 1D) of fecal pellets in *Tac1*^*-/-*^ compared to WT mice.

Although SP can also function as a secretagogue, inducing intestinal ion transport, no significant changes in short-circuit current (Isc) (S1B Fig), conductance (G) (S1C Fig), or macromolecular permeability indicated by the luminal to serosal translocation of 4 KDa FITC-Dextran (S1D Fig) were observed in non-infected WT compared to *Tac1*^*-/-*^ mice. The physiological response to carbachol (ΔIsc) was also not different in WT versus *Tac1*^*-/-*^ *mice* (S1E Fig). Infection-induced colonic histopathology was assessed using H&E-stained colonic tissue sections (Fig 1E), and crypt hyperplasia was assessed. As expected, *C. rodentium* infection-induced colonic crypt hyperplasia, indicated by significantly increased crypt length in WT mice compared to non-infected controls (Fig 1F). A significant reduction in crypt length was observed in infected *Tac1*^*-/-*^ compared to WT mice (Fig 1F). The putative role of MrgprA3 as an alternative non-canonical SP receptor was also assessed by administration of the antagonist Dictamnine. Mice infected with *C. rodentium* and treated with Dictamnine exhibited equivalent bacterial burden and histopathology compared to infected vehicle treated controls (S1F&G Fig). As constitutive Tac1 deficiency could induce compensatory changes in other neuropeptide pathways, we assessed expression of the neuropeptides *Grp, Nmu, Npy, Pdyn, Sst,* and *Vip* in whole colonic tissue. Infection with *C. rodentium* generally reduced expression of several neuropeptide transcripts in WT mice, whereas responses in *Tac1*^*-/-*^ mice were more variable, with maintained *Vip* and *Pdyn* expression during infection (S2 Fig).

#### Tac1-deficient mice exhibit reduced innate immune cell responses to *C. rodentium.*

**Fig 2:**
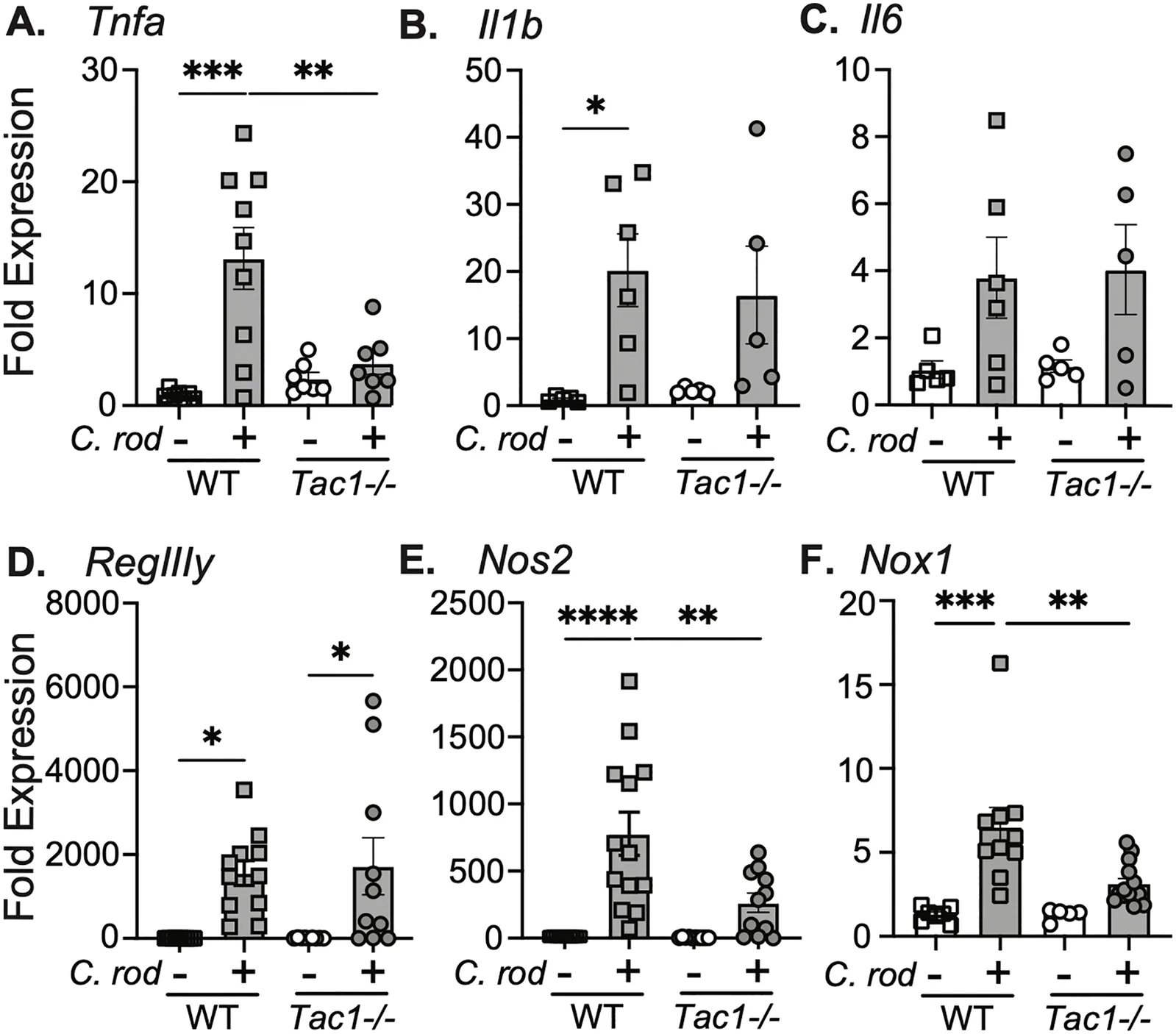
Deficiency in *Tac1* abrogates expression of select host-protective genes. Full-thickness colonic tissues from uninfected control or *C. rodentium* infected WT and *Tac1*^*-/-*^ mice were assessed for mRNA expression of the proinflammatory cytokines *Tnfa* **(A)**, *Il1b* **(B)**, and *Il6* **(C)**. Expression of *RegIIIy* **(D)**, *Nos2* **(E)**, and *Nox1* **(F)** mRNA was assessed using qRT-PCR. Data are presented from individual mice with mean ± SEM, *P < 0.05, ** P < 0.005, ***P < 0.001, one-way ANOVA followed by post-hoc analysis with Tukey’s multiple comparison test.

Infection-induced colonic inflammation was assessed in uninfected and infected WT and *Tac1*^*-/-*^ mice. Although *C. rodentium* infection significantly increased expression of the proinflammatory cytokine *Tnfa* in WT mice, this was significantly reduced in *Tac1*^*-/-*^ mice 10 days post-infection (Fig 2A). Despite this, there was no effect of *Tac1* deficiency on the expression of *Il1b* or *Il6* in uninfected or infected mice (Fig 2B & C). As expected, *C. rodentium* infection significantly increased expression of *RegIIIγ* irrespective of genotype (Fig 2D). In stark contrast, *Nos2* and *Nox1* expression were significantly upregulated at 10 dpi only in WT, but not *Tac1*^*-/-*^ mice (Fig 2E & F). We further used qPCR to assess FACS cell populations in naïve and *C. rodentium-*infected WT mice to understand cellular sources of the neuropeptide better. Using this approach, we found that compared to the dorsal root ganglia, *Preprotachykinin-1 (Tac1)* expression was exceedingly low in intestinal epithelial cells (IEC), colonic T-cells, neutrophils, as well as blood and lymphatic endothelium (S3 Fig).

#### Reduced recruitment of innate effector immune cells in *Tac1*^*-/-*^ mice during *C. rodentium* infection.

**Fig 3:**
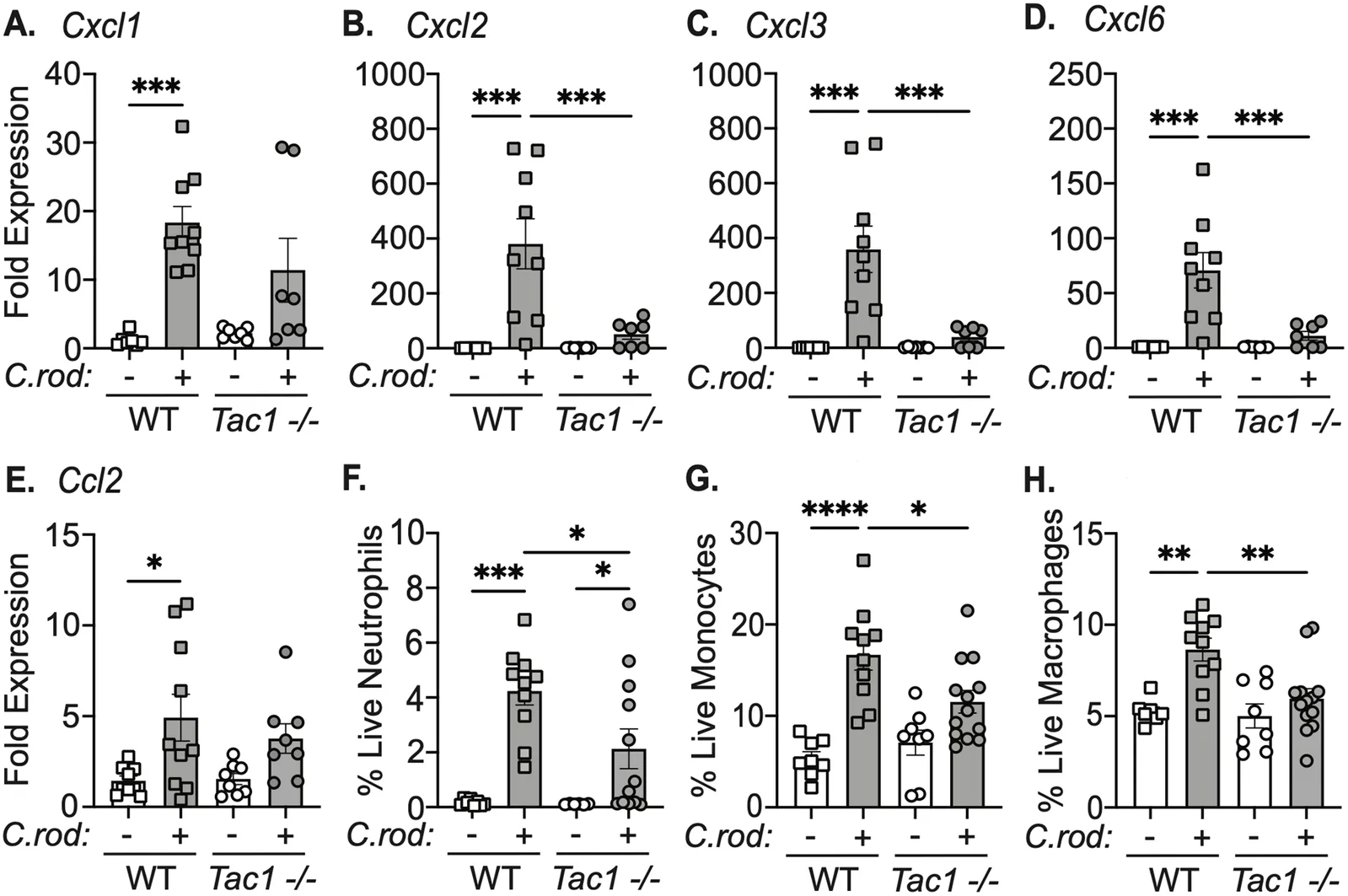
Decreased innate immune cell recruitment in *Tac1*^*-/-*^ mice during C*. rodentium* infection. Expression of the chemokines, *Cxcl1* **(A)**, *Cxcl2* **(B)**, *Cxcl3* **(C)**, *Cxcl6* **(D)**, and *Ccl2* **(E)** in the colon of uninfected control or *C. rodentium-infected* (10 dpi) WT and *Tac1*^*-/-*^ mice were assessed. Flow cytometry was conducted on single cell suspensions from the colonic lamina propria to determine the frequency of live neutrophils (CD45^+^ CD3^-^ Ly6G^+^) **(F)**, monocytes (CD45^+^ CD3^-^ Ly6G^-^ CD64^hi/low^ Ly6C^+^) **(G)**, and macrophages (CD45^+^ CD3^-^ Ly6G^-^ CD64^hi^ Ly6C^-^) **(H)**. Data presented as individual mice with mean ± SEM, *P < 0.05, ** P < 0.005, ***P < 0.001, one-way ANOVA followed by post-hoc analysis with Tukey’s multiple comparison test.

We sought to further characterize the innate immune response during *C. rodentium* infection in *Tac1*^*-/-*^ compared to WT mice. As neutrophils and monocytes are critical to host protection, we first assessed the expression of chemokines known to regulate homing of these cell populations. Although *Cxcl1* was not reduced significantly, significant reductions in *Cxcl2*, *Cxcl3*, and *Cxcl6* were observed in infected *Tac1*^*-/-*^ compared to WT mice at 10 dpi (Fig 3A-D). Expression of *Ccl2* increased significantly only in WT mice infected with *C. rodentium* (Fig 3E).

To understand the effect of these changes in chemokine expression on the recruitment of innate immune cells, we performed immunophenotyping of the colonic lamina propria from uninfected or *C. rodentium*-infected mice at 10 dpi (gating strategy, S4 Fig). As expected, infection significantly increased the frequency of neutrophils in WT and *Tac1*^*-/-*^ mice compared to their respective uninfected controls. Despite this, *C. rodentium* infected *Tac1*^*-/-*^ mice exhibited significant reductions in neutrophil recruitment compared to WT controls 10 dpi (Fig 3F). Additionally, the frequency of colonic monocytes and macrophages was significantly reduced at 10 dpi in *Tac1*^*-/-*^ compared to WT mice (Fig 3G).

#### Adaptive immune responses to enteric bacterial infections are reduced in *Tac1*^*-/-*^ mice.

**Fig 4:**
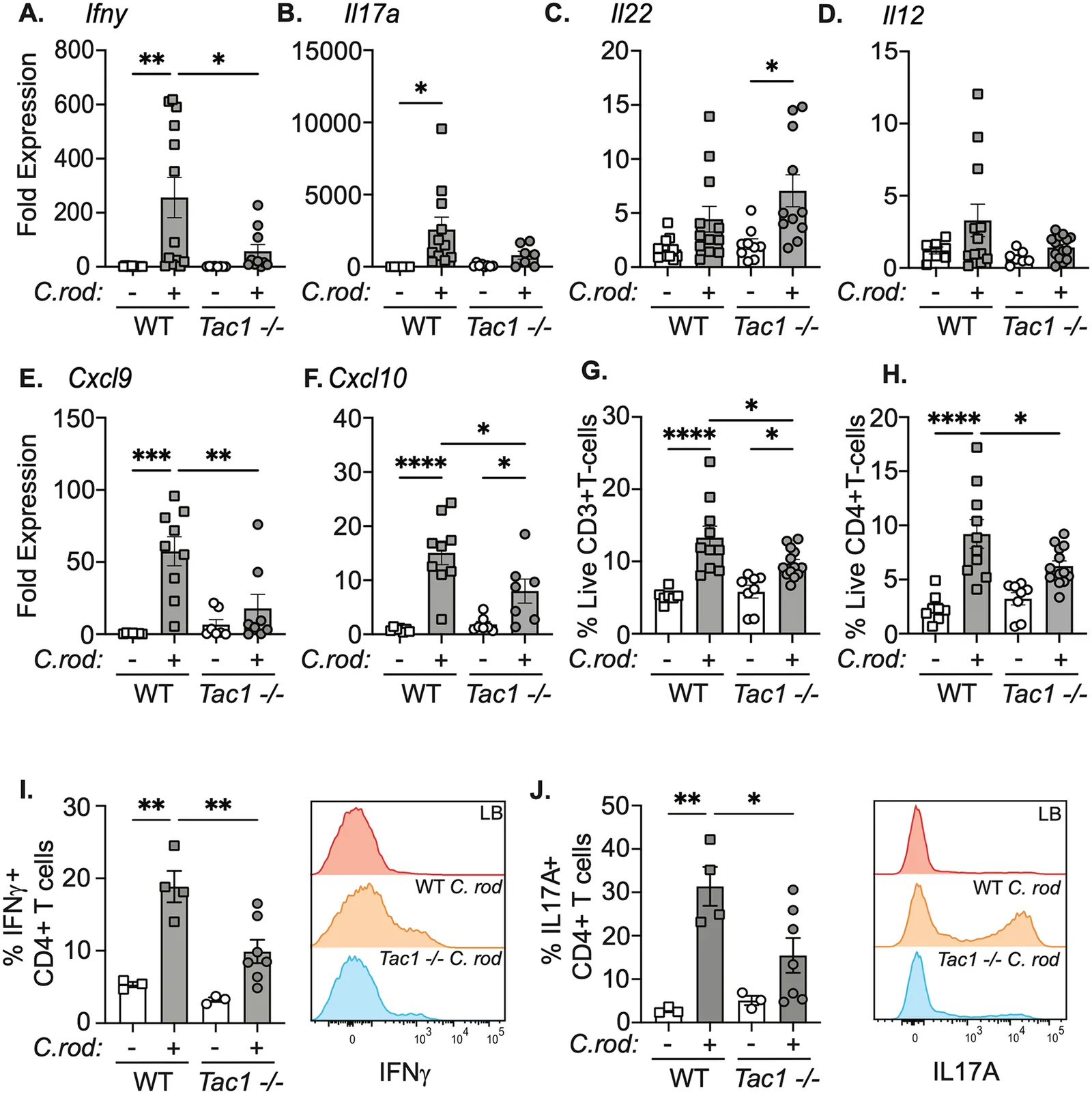
T-cell recruitment is diminished in *C. rodentium*-infected *Tac1*^*-/-*^ mice. Expression of the cytokines *Ifn*γ **(A)**, *Il17a* **(B)**, *Il22* **(C),** and *Il12* **(D)** mRNA in the colon of uninfected or infected WT and *Tac1*^*-/-*^ mice was evaluated by qRT-PCR. Quantification of the chemokines *Cxcl9* **(E)**, and *Cxcl10* **(F)** mRNA in the colon of sham or infected WT and *Tac1*^*-/-*^ mice 10 dpi. Flow cytometry was performed on single-cell suspensions from the colonic lamina propria to identify the frequency of T-cells (**G.** live, CD45^+^ CD3^+^), and CD4 + T-cells (**H.** live CD45^+^ CD3^+^ CD4^+^). Intracellular cytokine staining on lamina propria lymphocytes was performed to reveal IFNγ **(I)** and IL17A **(J)** T-cells (live CD45^+^ CD3^+^ CD4^+^). Data are presented from individual mice with mean ± SEM, *P < 0.05, ** P < 0.005, ***P < 0.001, one-way ANOVA followed by post-hoc analysis with Tukey’s multiple comparison test.

Given the importance of adaptive immunity in the host response to *C. rodentium* infection, we assessed the expression of chemokines and cytokines, and recruitment of T-cells in WT and *Tac1*^*-/-*^ mice. Expression of *Ifn*γ was significantly increased in the colon at 10 dpi of WT mice compared to uninfected controls, but this response was significantly diminished in infected *Tac1*^*-/-*^ mice (Fig 4A). Similarly, *Il17a* was significantly increased only in WT mice, but not *Tac1*^*-/-*^ mice. Increased expression of *Il22* was observed in infected vs uninfected mice irrespective of genotype (Fig 4B & C). The attenuated IFNγ production during infection further prompted us to evaluate *Il12* expression, which was not significantly increased during infection in WT or *Tac1*^*-/-*^ mice (Fig 4D). As chemokines are a critical mechanism to recruit T-cells into the colon during enteric bacterial infection, we assessed the expression of common T-cell chemokines. Significant attenuation of the T-cell chemokines *Cxcl9* and *Cxcl10* were observed in *Tac1*^*-/-*^ versus WT-infected mice (Fig 4E & F). To assess the potential functional implications of reduced chemokine and cytokine expression, flow cytometry was performed to assess T-cell recruitment. During *C. rodentium* infection, *Tac1*^*-/-*^ mice had significantly reduced CD4 + T-cells in the lamina propria compared to WT controls (Fig 4G & H). Using intracellular cytokine staining, we found significantly reduced IL-17A and IFNγ expression in live CD4 + T-cells isolated from the lamina propria in *Tac1*^*-/-*^ vs WT mice. We further assessed if a reduced ability to produce IFNγ was due to an intrinsic defect in T-cells from *Tac1*^*-/-*^ mice. *In vitro* differentiation revealed no significant difference in the ability to polarize T-cells from the MLN under Th1 conditions (S4B Fig). To determine whether altered humoral immunity was associated with the reduced bacterial burden in *Tac1*^*-/-*^ mice, we assessed total and *C. rodentium*-reactive responses for selected immunoglobulin isotypes. Infected WT mice exhibited increased serum *C. rodentium*-reactive IgA, and IgG3 compared to *Tac1*^*-/-*^ mice. No significant differences were observed in *C. rodentium*-reactive IgG1, IgG2b, IgG2c, or IgGM between infected WT and *Tac1*^*-/-*^ mice (S5A Fig). No significant differences were observed in the total serum concentrations of the measured immunoglobulin isotypes. Together, these findings do not support a broadly enhanced class-switched antibody response as the explanation for reduced bacterial colonization in Tac1-deficient mice at this stage of infection (S5B Fig).

## Discussion

Mucosal immune responses in the GI tract are tightly regulated processes. Although rapid and robust responses are required at this interface with the external environment to prevent potential pathogen entry, responses to dietary and commensal microbiota-derived substances can be deleterious. Host responses to enteric bacterial pathogens such as *C. rodentium* require successive activation of resident and recruited immune cell populations. Effective coordination of these processes occurs through numerous overlapping mechanisms, including bidirectional communication between the nervous and immune systems [6,29–31]. This functionality includes the detection of cytokines by specialized sensory neurons within the affected tissues of immune organs [16,32]. During *C. rodentium* infection, the polymodal nociceptive protein TRPV1 and sensory neurons play a critical role in the recruitment of neutrophils to the infected colon [15,16]. It is well established that these neurons can release neuropeptides, such as SP, locally in response to noxious stimuli, thereby triggering neurogenic inflammation [33]. The molecular underpinnings of this response are due to activation of SP receptor on diverse cell types, including endothelial cells, to induce vascular permeability and increase cellular adhesion molecule expression. We have previously demonstrated that the SP receptor plays a critical role in regulating colonic inflammation during *C. rodentium* infection [17]. Complicating the interpretation of the role of SP receptor signaling in mediating host protection, other ligands, such as Hemokinin-1 (Tac4), can activate the SP receptor [34]. With this complexity in mind, we sought to provide a more comprehensive picture of the role of the neuropeptides encoded by *Tac1* in regulating host defense. Here, we demonstrate that specific aspects of the immune response to *C. rodentium* are altered in *Tac1*^*-/-*^ mice. Infection of *Tac1*^*-/-*^ mice with *C. rodentium* significantly reduced bacterial burden and fecal shedding compared to WT mice. These data were in keeping with our prior studies using pharmacological antagonists of the SP receptor [17]. Given the known roles of SP as a positive regulator of colonic motility [35], we considered whether fecal pellet output as a measure of distal colonic motility was altered in *Tac1*^*-/-*^ compared to WT mice. This observed reduction in bacterial burden in *Tac1*^*-/-*^ mice was not due to increased colonic motility, as we observed significantly reduced fecal pellet output compared to WT controls. These data are also in keeping with observations of reduced fecal output with SP receptor antagonism [17]. As SP has long been described as a secretagogue that increases the secretory state of the GI tract [36,37], we performed Ussing chamber studies with colonic tissues from WT and *Tac1*^*-/-*^ mice. We noted no significant differences in the baseline or evoked Isc, conductance, or macromolecular permeability in *Tac1*^*-/-*^ mice*,* suggesting differences in host immune responses were not due to altered baseline intestinal physiology.

As the immune response to *C. rodentium* is well-appreciated to induce colonic epithelial cell hyperplasia [4,38], we assessed this host response to infection. Reduced bacterial burden in *Tac1*^*-/-*^ mice compared to WT was associated with reduced colonic crypt hyperplasia. In WT mice, these structural changes are a consequence of inflammation and pro-inflammatory cytokines increasing epithelial cell proliferation and the number of non-differentiated epithelial cells along the crypt axis [39]. Infection-induced increases in IFNγ-IFNγ receptor signaling are a known driver of crypt hyperplasia, as infection of IFNγ receptor-deficient mice fail to induce crypt hyperplasia [38,40]. Our data showing reduced expression of pro-inflammatory cytokines in infected *Tac1*^*-/-*^ mice suggests that reduced histopathology is due to reduced inflammation. Here we demonstrate that *Tac1*^*-/-*^ mice have significantly reduced IFNγ expression and recruitment of CD4 + IFNγ + T-cells, and consequently reduced IFNγ-regulated genes, including *Nos2 [*41*]* and *Nox1*, during *C. rodentuim* infection. While the enzymes encoded by these genes are typically regarded as host-protective, Nos2^-/-^ mice exhibit reduced bacterial clearance without increased morbidity or mortality during *C. rodentium* infection [41,42]. More recently, the production of H_2_O_2_ has also been shown to be host maladaptive, supporting the proliferation of enteric bacterial pathogens, including *C. rodentium* [43*]*. Although elevated colonic nitrate due to increased Nos2 activity supports the growth of *Salmonella enterica* and select commensal bacteria, *C. rodentium* does not use nitrate as a terminal electron acceptor in support of metabolism and growth. However, inflammation and the ensuing colonic oxygenation increase *C. rodentium* proliferation [44]. These data suggest that the immune response to *C. rodentium* and colonic inflammation can be host maladaptive. Reduced IFNγ and inflammation in *Tac1*^*-/-*^ mice therefore suggest that, in WT mice, these neuropeptides enhance inflammatory processes that support pathogen growth. These data are in keeping with our prior observations, where antagonism of the SP receptor reduced bacterial burden, IFNγ, and IFNγ-dependent gene expression during *C. rodentium* infection. Together, these data suggest the enhancement of IFNγ production by a SP-SP receptor signaling axis during enteric bacterial infection, and that this response can evoke host maladaptive responses. Also of note, we observed reduced *Tnfa* in infected *Tac1*^*-/-*^ mice, a feature not observed in our prior SP receptor antagonist studies [17]. These results may suggest a possible role for neurokinin A, as the other peptide encoded by the mouse *Tac1* gene, or that a non-canonical receptor could be engaged. SP can also activate Mas-related G protein-coupled receptors (Mrgprs), inducing a variety of biological effects, ranging from activation of nociceptors to mast cell degranulation [45,46]. Using Dictamnine as a pharmacological antagonist of MrgprsA3, we noted no difference in bacterial burden or pathology during enteric bacterial infection. These results, however, do not exclude a role for other Mrgprs subtypes during *C. rodentium* infection. Mast cells have been demonstrated to exert an antibacterial effect in *C. rodentium* infection [47], and Mrgprs-mediated activation of mast cells limits skin infection [30]. Whether SP-induced Mrgprs signaling similarly modulates mast cell-dependent host responses in the gut remains unknown.

Substance P exerts additional control over the immune response during infection, with *Tac1*^*-/-*^ mice exhibiting reduced expression of select chemokines that result in neutrophil and T-cell recruitment. Significant reductions in these chemokines were further matched by reduced recruitment of these cell types to the infected colon of *Tac1*^*-/-*^ mice compared to WT. Once again, these reductions in T-cell recruitment mirror the effects observed in infected SP receptor antagonist-treated mice that we reported previously [17]. These findings suggest that SP receptor signaling enhances the recruitment of immune cell populations through different mechanisms, each of which could be targeted for modulation. For example, SP-induced activation of blood endothelial cells would have different effects on the development of an immune response compared to activation of stromal cells. As part of the unique biological outcomes, not only are the responding cell populations important, but the source of SP, and thus the location of the rapidly clearing peptide, is a critical factor to consider as well. Although many sources of SP in the intestine have been previously identified, including immune cells, stroma, and intestinal epithelial cells [24,33,48], we found low levels of *Tac1* expression in these cell types from uninfected and *C. rodentium-infected* mice compared to sensory neurons of the dorsal root ganglia. It is important to note that these data do not exclude the potential for rare but biologically significant sources of SP in these populations; rather, they may indicate that, in the bulk population or in each cell type, expression is very low. Indeed, this would likely include the reported SP-producing enteroendocrine cells [24]. The cell types that produce biologically relevant amounts of SP during *C. rodentium* infection remains an open question that should be addressed using conditional knockout mice in future studies.

An important consideration when interpreting the *Tac1*^*-/-*^ phenotype is the potential for adaptation in other neuropeptide pathways. Initial characterization of *Tac1*^*-/-*^ mice found broadly preserved sensory neuron organization [49], whereas subsequent analysis of the spinal dorsal horn identified selective reductions in GRP, CGRP, and NPY immunoreactivity [50]. These findings indicate that constitutive Tac1 deficiency can alter individual peptidergic markers but does not establish broad compensatory upregulation of alternative neuropeptide pathways. Consistent with this, our analysis of whole colon identified selective differences in Vip and Pdyn regulation during infection, without coordinated upregulation across the assayed neuropeptide genes. Together, our data demonstrate a significant role for neuropeptides encoded by *Tac1* in the host response during enteric bacterial infection. We further speculate that modulation of this signaling axis may provide a previously unappreciated mechanism to limit host maladaptive inflammation without impinging upon bacterial clearance.

## Supporting information

S1 Fig Substance P and Non-Canonical Receptors in Infection and Intestinal Physiology. Fecal bacterial burden was determined at Day 4, Day 14, and Day 30 (A). Colonic physiology including short circuit current “Isc” (B), conductance “G” (C), macromolecular permeability (D), and evoked Isc response to carbachol (E) in uninfected WT and *Tac1*^*-/-*^ mice was assessed by Ussing chamber. Involvement of non-canonical substance P receptor MrgprA3 was determined by bacterial enumeration in feces and distal colon 10 dpi (F) as well as H&E staining of distal colon (G) in mice treated with Dictamnine (2.5 mg/Kg, i.p) or Vehicle (5% DMSO in PBS). Data are presented from individual mice with mean ± SEM, Student’s two-tailed T-test, or ANOVA (F), * P < 0.05.
(TIFF)

S2 Fig Selective changes in colonic neuropeptide expression during *C. rodentium* infection in Tac1-deficient mice. Full-thickness colonic tissues were collected from uninfected and *C. rodentium*-infected WT and *Tac1*^*-/-*^ mice at 10 days post-infection. Relative mRNA expression of *Grp*, *Nmu*, *Npy*, and *Pdyn*, *Sst*, and *Vip*, was assessed by qRT-PCR. Data are presented for individual mice with mean ± SEM. Statistical significance was determined by two-way ANOVA followed by Tukey’s multiple-comparison test. *P < 0.05, **P < 0.005, ***P < 0.001, ****P < 0.0001; ns, not significant.
(TIFF)

S3 Fig *Tac1* expression in FACS-sorted cell populations. Single cell suspensions from the colon of uninfected and infected WT mice (10 days post-infection) were subjected to FACS followed by qPCR to detect *Tac1* mRNA expression in blood endothelial cells, lymphatic endothelial cells, T-cells, and neutrophils. The expression of *Tac1* was normalized to *Tac1* expression from WT dorsal root ganglia. Intestinal epithelial cells were obtained by agitation in EDTA-containing media. Data are presented from individual mice with mean ± SEM, one-way ANOVA followed by post-hoc analysis with Tukey’s multiple comparison test.
(TIFF)

S4 Fig Flow cytometry gating analysis and *in vitro* T-cell differentiation assays. Gating strategy used to enumerate the indicated cell populations (A) from the colon of uninfected or 10 days post-*C. rodentium* infection in WT or *Tac1*^-/-^ mice. The ability of mesenteric lymph node T-cells from *Tac1*^*-/-*^ mice or WT to differentiate and produce IFNγ was assessed by ELISA following *in vitro* Th1 differentiation and stimulation (B). Data are presented from individual mice with mean ± SEM, *P < 0.05, ** P < 0.005, ***P < 0.001, one-way ANOVA followed by post-hoc analysis with Tukey’s multiple comparison test.
(TIFF)

S5 Fig Total and *C. rodentium*-reactive IgG responses in WT and *Tac1*^*-/-*^ mice. Serum was collected from WT and *Tac1*^*-/-*^ mice 10 days post-infection with *C. rodentium*. Total IgG (A) and *C. rodentium*-reactive IgG (B) were quantified by ELISA. Data are presented for individual mice with mean ± SEM. Group comparisons used two-way ANOVA with Geisser-Greenhouse correction and Sidak’s multiple comparisons test, P ≤ 0.05. ns, not significant.
(TIFF)
